# Explained variation of excess hazard models

**DOI:** 10.1002/sim.7645

**Published:** 2018-04-06

**Authors:** Camille Maringe, Maja Pohar Perme, Janez Stare, Bernard Rachet

**Affiliations:** ^1^ Cancer Survival Group London School of Hygiene and Tropical Medicine Keppel Street London WC1E 7HT UK; ^2^ Department of Biostatistics and Medical Informatics University of Llubljana Vrazov trg 2 SI‐1000 Ljubljana Slovenia

**Keywords:** excess hazard models, explained variation

## Abstract

The availability of longstanding collection of detailed cancer patient information makes multivariable modelling of cancer‐specific hazard of death appealing. We propose to report variation in survival explained by each variable that constitutes these models. We adapted the ranks explained (RE) measure to the relative survival data setting, ie, when competing risks of death are accounted for through life tables from the general population. RE is calculated at each event time. We introduce weights for each death reflecting its probability to be a cancer death. RE varies between −1 and +1 and can be reported at given times in the follow‐up and as a time‐varying measure from diagnosis onward. We present an application for patients diagnosed with colon or lung cancer in England. The RE measure shows reasonable properties and is comparable in both relative and cause‐specific settings. One year after diagnosis, RE for the most complex excess hazard models reaches 0.56, 95% CI: 0.54 to 0.58 (0.58 95% CI: 0.56–0.60) and 0.69, 95% CI: 0.68 to 0.70 (0.67, 95% CI: 0.66–0.69) for lung and colon cancer men (women), respectively. Stage at diagnosis accounts for 12.4% (10.8%) of the overall variation in survival among lung cancer patients whereas it carries 61.8% (53.5%) of the survival variation in colon cancer patients. Variables other than performance status for lung cancer (10%) contribute very little to the overall explained variation. The proportion of the variation in survival explained by key prognostic factors is a crucial information toward understanding the mechanisms underpinning cancer survival. The time‐varying RE provides insights into patterns of influence for strong predictors.

## 1 INTRODUCTION

Complex, multivariable modelling of time‐to‐event data is easily accessible through user‐friendly specific commands in common statistical software.^1^, ^2^, ^3^, ^4^ In such models, the effects of prognostic factors on hazard of death are modelled and estimated. Possible non‐linearity and time dependence of their effects can be incorporated. The model gives the usual estimates of effect and *P*‐values, but often the estimation of survival for the cohort is the metric of choice.

Datasets in population‐based research contain information on virtually all patients in a given area or country for a given period of time: it can represent such large numbers that statistical significance does not bring much information on the relative importance of prognostic factors. A measure of explained variation does not aim at providing information on how well a model fits the data at hand but provides information on how much of the variation in survival between records is explained by the model, and hence by the prognostic factors that compose the model.

Although survival models do not carry good prediction properties, there is a number of measures proposed for evaluating their prognostic characteristics^5^ by ways of measures of prediction accuracy,^6^ discrimination potential,^7^ and the proportion of variation explained.^8^ Most measures have been designed in the context of the Cox model,^9^ widely used in traditional survival analyses or clinical trials. However, when focussing on survival from a disease, eg, cancer, survival analysis needs to account for competing risks of death. In the population‐based cancer survival context, the exact cause of death of patients is unknown or considered unreliable. In this context, we rely on the relative survival data setting, in which the hazard of death from the cancer, or excess hazard, is estimated by comparing the overall mortality of the cancer patients to their expected mortality provided by life tables built for the general population from which the cancer patients come.^10^ The effects of explanatory variables on the excess hazard can be modelled using various excess hazard models.^3^, ^11^ Net survival, the survival of the cohort of cancer patients, cancer being the only cause of death, can be derived from such models, providing these are well specified. The assumption of informative censoring is replaced by a more plausible assumption of independence of the forces of mortality, providing the effects of the variables stratifying the life tables, such as sex, age, region, deprivation, and ethnicity, are adjusted for in the model.^12^

In this paper, we adapt a measure of explained variation, ranks explained (RE),^13^ to the context of excess hazard models in the relative survival data setting. We address challenges related to the specificities of that setting and the excess hazard modelling, while the interpretation of the adapted RE is kept as simple as with the original RE measure. This is exemplified by an extensive illustration using population‐based cancer registry data on patients diagnosed with colon or lung cancer in England.

The next section summarises the characteristics of the measure of explained variation, RE, then presents the excess hazard models and how RE was adapted to the relative survival data setting. In a third section, we describe the design of our simulation‐based analyses aimed at exploring the features of RE. The following section presents an application based on colon and lung cancer patients in England. The discussion wraps up the main advantages and limitations of the measure proposed.

## 2 METHODS

### 2.1 The RE measure in the overall survival setting

The RE measure, standing for “ranks explained”, was introduced by Stare et al.^13^ It aims at providing a measure of the variation in the ranks observed in survival‐time data explained by a given model. It can be viewed as a generalisation of the C‐index.^14^ It satisfies the following list of criteria:

1. Applicability to multiple end‐point survival
2. Facility to incorporate time‐varying and/or dynamic covariates and/or time‐dependent effects
3. Model‐free interpretation on a well‐understood scale, to allow comparison between non‐nested models
4. Applicability to both parametric and semiparametric models
5. Consistency under general independent censoring mechanisms, including intermittent missingness and delayed entry or truncation

Some of these points, particularly (2), (3), and (5), make the measure appealing to the excess hazard context.

Technically, the sum of the variation in ranks, explained by the model is compared with the sum of the total variation in ranks there is to explain. The “unit” is the rank that each record is given at each failure time *t*_*i*_, ie, the predicted position at which the record under observation will fail among all observations that have yet to fail (observations in the *risk set* *R*_*i*_). The total variation is viewed as the difference between the ranks allocated under a “null model” (*r*_*i,null*_), and the ranks allocated under a “perfect model” (*r*_*i,perfect*_), ie, the record that fails is always given rank 1:

$$\begin{array}{l} r_{i,\text{null}}=\frac{\left(k+1\right)}{2}\;\forall k\;\in \;R_{i,}\;i.\;e.\;t_{k}\;>\;t_{i}\\ \;r_{i,\text{perfect}}=1. \end{array}$$

We define the “null model” as a model in which all records that have not yet failed are given the same mean rank: it corresponds to a scenario in which one would lack information regarding the expected time to failure of the individuals in the risk set, and all individuals would therefore have the same probability to fail next.

The variation that is explained by a proposed model is the difference between the ranks allocated under a “null model” and the ranks that are allocated under the proposed model (*r*_*i,model*_).

$$r_{i,\text{model}}=1+\sum_{k\in R_{i}}l_{\lambda_{k\left(t_{i}\right)}>\lambda_{i\left(t_{i}\right)}}$$

Where $\lambda_{k\left(t_{i}\right)}$ and $\lambda_{i\left(t_{i}\right)}$ are the hazards for patients *k* and *i*, respectively, at patient's *i* time of failure *t*_*i*_.

The final statistic sums these differences over all individual failure times so that the statistic is defined, in the case of single‐event survival data by:

(1) $$\mathrm{RE}=\frac{\sum_{i}\left(r_{i,\text{null}}-r_{i,\text{model}}\right)}{\sum_{i}\left(r_{i,\text{null}}-r_{i,\text{perfect}}\right)}.$$

Through censoring patients leave the cohort. In order for those who stay in the cohort to be representative of those who left, we weight records that are more likely to have missing observed failure time. Typically, the weights are the reverse Kaplan Meier estimates ( $\frac{1}{\hat{G_{\mathrm{it}}}}$), in the case of survival data with right censoring.^5^, ^15^ The delta method is also used to provide a formulation for the variance of RE. Full details can be found in Stare et al.^13^

(2) $$\mathrm{RE}=\frac{\sum_{i}\int_{0}^{\tau }\frac{1}{\hat{G_{\mathrm{it}}}}*\left(r_{i,\text{null}}\left(t\right)-r_{i,\text{model}}\left(t\right)\right)dN_{i}\left(t\right)}{\sum_{i}\int_{0}^{\tau }\frac{1}{\hat{G_{\mathrm{it}}}}*\left(r_{i,\text{null}}\left(t\right)-r_{i,\text{perfect}}\left(t\right)\right)dN_{i}\left(t\right)}$$

In Equations (1) and (2), the sum is by default over all observations *N* that fail in the sample. It is also of interest to estimate instantaneous measures of explained variation, termed *local RE*, for which the sum is made over the *x* records that fail around each successive observed failure times throughout the entire follow‐up. The value of *x* depends on the cancer, but the illustrations presented here used a window of 20 failures.

(3) $$\text{localRE}=\frac{\sum_{i}\int_{t-x/2}^{t+x/2}\frac{1}{\hat{G_{\mathrm{it}}}}*\left(r_{i,\text{null}}\left(t\right)-r_{i,\text{model}}\left(t\right)\right)dN_{i}\left(t\right)}{\sum_{i}\int_{t-x/2}^{t+x/2}\frac{1}{\hat{G_{\mathrm{it}}}}*\left(r_{i,\text{null}}\left(t\right)-r_{i,\text{perfect}}\left(t\right)\right)dN_{i}\left(t\right)}$$

### 2.2 The excess hazard model

Net survival is the survival that would be observed in our population of cancer patients, had cancer been the only possible cause of death.^16^ Net survival can be estimated in the cause‐specific setting or in the relative survival setting. The main difference between the 2 settings is the knowledge of the cause of death.

**Figure 1:**
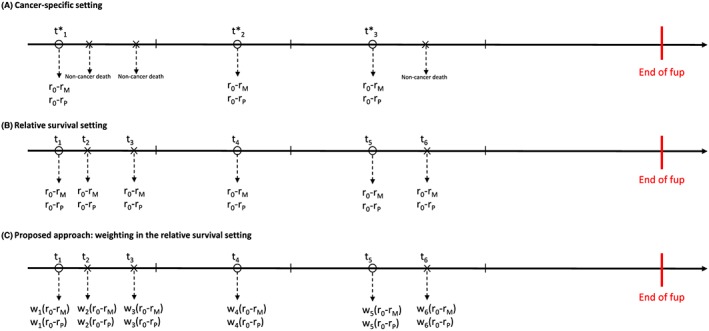
Calculation of RE in different settings (A) Cancer‐specific setting (B) Relative survival setting (C) Proposed approach: Weighting in the relative survival setting. ◯ time of cancer death; X time of non‐cancer death; | time of censoring; r_M_: Rank as estimated from the model‐derived hazard of death; r_0_: Average rank of the records in the risk set; r_P_: 1; w_i_: probability of cancer death [Colour figure can be viewed at http://wileyonlinelibrary.com]

In the cause‐specific setting, the exact cause of death is known, and the failure indicator reflects whether the patient dies from his/her cancer (failure is coded 1), did not die (failure is 0), or died from a cause other than cancer (failure is 0 or 2). It is straightforward to adapt RE to cause‐specific survival models: the only difference is that RE is evaluated at each cancer death rather than each death (see Figure 1A).

In the relative survival setting, cause of death is not available or not deemed reliable; therefore, population life tables are used in the modelling of excess mortality to adjust for mortality due to other causes, also termed expected or background mortality. Population life‐tables reflect the pattern of survival of the general population, from which the cancer patients are drawn. In population‐based cancer survival, the relative survival setting is the setting of choice for the estimation of net survival through excess hazard modelling.

We aim that RE gives a measure of how much of the *cancer* survival variation observed between individuals is explained by a specific excess hazard model: we remove the impact of other causes and isolate the effects of potential additional variables on cancer mortality.

### 2.3 RE measured from an excess hazard model

- Weights

In the same way that consistent estimators of net survival can be obtained in both relative survival and cause‐specific settings, we want RE calculated in both settings to agree. In the cause‐specific setting, RE is evaluated only at times of cancer deaths (Figure 1A). By contrast, the relative survival setting uses all failure times regardless the cause of death (Figure 1B) for which RE needs to be adjusted (Figure 1C).

Therefore, we propose to weight each event time with quantities reflecting the probability that the event is happening due to the cause of interest at the time considered. We therefore consider

(4) $$w_{i}=p\left(\left.dN_{E_{i}}\left(t\right)=1\right|\;dN_{i}\left(t\right)=1\right)$$

where $N_{E_{i}}\left(t\right)$ is the counting process associated with the cause of interest, and *N*_*i*_(*t*) is the all‐cause counting process.

We define the weights as the ratio of the excess mortality due to cancer *λ*_*E*_, over the sum of the excess and expected (population, *λ*_*P*_) mortality.^17^ Both hazards are estimated at the time of death.

(5) $$w_{i}=w\left(t_{i}\right)=\frac{\lambda_{E_{i}}\left(t_{i}\right)}{\lambda_{E_{i}}\left(t_{i}\right)+\lambda_{P_{i}}\left(t_{i}\right)}$$

Take the practical example of the cause‐specific setting: if we were to use weights, differences in ranks would be evaluated at times at which patients are censored due to death from other causes, but their weight, hence contribution, would be 0, because the probability that the event is a cancer death is null. To mirror this in the relative survival setting, weights would tend to 0 when the probability of cancer death is highly unlikely, and weights would tend to 1 when the probability of cancer death is highly likely.

We want to show that the total number of cancer events can be estimated by the sum of weights *w*_*i*_. By law of total probabilities, we have

(6) $$p\left(dN_{E_{i}}\left(t\right)=1\right)=p\left(\left.dN_{E_{i}}\left(t\right)=1\right|\;dN_{i}\left(t\right)=1\right)*p\left(dN_{i}\left(t\right)=1\right)+p\left(\left.dN_{E_{i}}\left(t\right)=1\right|\;dN_{i}\left(t\right)=0\right)*p\left(dN_{i}\left(t\right)=0\right)$$

Because $p\left(\left.dN_{E_{i}}\left(t\right)=1\right|\;dN_{i}\left(t\right)=0\right)=0$, and *dN* variables are binomial variables, if one sums Equation (6) over individuals and event times, after changing the order of summation and expectation, one gets:

(7) $$E\left(\sum_{i=1}^{n}\sum_{t_{k}}dN_{E_{i}}\left(t_{k}\right)\right)=E\left(\sum_{i=1}^{n}\sum_{t_{k}}w_{i}*dN_{i}\left(t_{k}\right)\right)$$

Given that *dN*_*i*_(*t*_*k*_) = 1 if and only if *t*_*i*_ = *t*_*k*_, Equation (7) can be written as follows:

(8) $$E\left(\sum_{i=1}^{n}\sum_{t_{k}}dN_{E_{i}}\left(t_{k}\right)\right)=\sum_{i=1}^{n}w_{i}$$

The total number of cancer events can thus be estimated by the sum of weights: depending on the quality of the approximation of the expected mortality hazard by the general population life tables and the excess hazard model to estimate cancer‐specific mortality, the sum of weights will approach the number of cancer deaths.

We define RE for excess hazard models, REw, as follows:

(9) $$\mathrm{REw}=\frac{\sum_{i}\int_{0}^{\tau }\;\frac{w_{\mathrm{it}}}{\hat{G_{\mathrm{it}}}}*\left(r_{i,\text{null}}\left(t\right)-r_{i,\mathrm{mo}d\mathrm{el}}\left(t\right)\right)dN_{i}\left(t\right)}{\sum_{i}\int_{0}^{\tau }\frac{w_{\mathrm{it}}}{\hat{G_{\mathrm{it}}}}*\left(r_{i,\text{null}}\left(t\right)-r_{i,\text{perfect}}\left(t\right)\right)dN_{i}\left(t\right)}$$

- Null models

In order to adapt RE to the relative survival setting, we kept the null model defined in Stare et al^13^ and presented in Section 2.1 above; additionally the use of weights reflects the probability that an event is the event of interest.

Nonetheless, alternative null models have been considered, which assume some features of the excess hazard model a “given”. For instance, we tested a null model that conveyed the life table information. The “null” rank (*r*_*i*,*null*_) attributed to each patient at each event time *t*_*i*_ was derived from decreasing expected (population) mortality rates measured at *t*_*i*_.

$$r_{i,\text{null}}=\mathrm{rank}\left(\lambda_{P_{i}}\left(t_{i}\right)\right)$$

It meant that for RE to be large, the effects of variables such as age, present in both the population life tables and the excess (cancer) hazard model, would need to have a different effect on the expected hazard and on the excess hazard. For example, age has a strong effect on both the expected mortality and the excess mortality; hence, both *r*_*i*,*null*_ and *r*_*i*,*model*_, respectively, are close to 1 for most patients i. Therefore, the individual difference *r*_*i*,*null*_ – *r*_*i*,*model*_ will be slightly positive only when *r*_*i*,*null*_ > *r*_*i*,*model*_, ie, when the effect of age on the expected hazard is smaller than the effect of age on excess mortality. A large β_*age*_ in the excess hazard model can therefore lead to a small overall RE: a result that is hard to interpret. Similarly, because some factors can cease to be discriminant for cancer survival years after diagnosis, the individual differences *r*_*i*,*null*_ – *r*_*i*,*model*_ become very negative so the local RE and even the overall RE could reach very negative values.

We also tested a null model that integrated the additive structure of the overall mortality into excess and expected hazards.

$$r_{i,\text{null}}=\mathrm{rank}\left(\lambda_{P_{i}}\left(t_{i}\right)+\lambda_{0}\left(t_{i}\right)\right)$$

Nonetheless, defining a model which only contained that structure with no further assumption was challenging, and was confusing the interpretation of RE.

We believe the null model presented and used in Stare et al^13^ in conjunction with our weighing remains the most relevant approach for the adaptation of the original RE to excess hazard models. Hence, *r*_*i*,*null*_ represents the mean rank of all observations in the risk set at time *t*_*i*_, reflecting a complete absence of knowledge on what observation will fail next. In this way, RE estimated through cause‐specific or relative survival settings using weights will have the same interpretation.

Several outputs can be defined from the explained variation measure:

- Time‐varying REw, REw(t):
  - it is considered as a function of follow‐up time and reports the values of REw cumulated up to given times
  - REw is the cumulative measure calculated over the entire follow‐up.

This is the main measure together with its variance or confidence interval.

- Local REw, an instantaneous measure of REw, measured using events happening between 2 pre‐defined times, possibly moving through the follow‐up.

This measure is exploratory, designed to investigate further specific explained variation patterns. It is advised to report smoothed curves of the local instantaneous REw values and time‐varying REw(t).

## 3 SIMULATIONS

We performed simulation studies to understand the properties of REw defined in the context of excess hazard models. The simulations also demonstrate the characteristics of REw such as the information it brings over the usual model outputs and how sensitive REw is to model mis‐specification.

### 3.1 Simulation strategy

- Data

We used information on 5809 breast (women) and 2418 lung (men and women) cancer patients diagnosed in England in 2000 with a valid stage at diagnosis. The potential maximum follow‐up was 8 years for each patient, to the 31^st^ December 2007, and information on their age, deprivation status, and stage at diagnosis was available. Due to passive follow‐up, no censoring happens prior to the end of follow‐up. Breast and lung cancers were chosen for their differing death patterns: 93% of lung cancer patients vs 30% of breast cancer patients die in the 8 years following diagnosis, and cancer deaths account for nearly 95% and around 60% of all deaths in lung and breast cancer respectively.

- Expected survival times

Expected survival times were simulated by extracting expected mortality rates, λ_P_, from sex‐specific, age‐specific, year‐specific, and deprivation‐specific life tables, defined at each month of age and every calendar month. Moving forward, at each anniversary day of diagnosis, patient records were merged to these life tables in order to get a patient‐specific expected mortality rate λ_P_ for that exact day. The survival time *u*, simulated for each patient from an exponential distribution with mean *λ*_*P*_, was compared with 1 month to determine the expected survival time: if *u* was always greater than 1, the patient over‐lived every month and was still alive at the end of the 8‐year follow up. The failure indicator equals to 1 when the subject dies (whatever the cause) or 0 otherwise.

- Parameters of the simulations

Fully parametric models were fitted on the log cumulative hazard scale^1^, ^11^ to model the excess hazard of death using the STATA command, stpm2. Model‐based information, such as the parameters of the baseline log‐cumulative excess hazard, and the estimated effect parameters, was used to simulate a thousand survival times (outcome) for each of the 5809 breast and 2418 lung cancer patients. We kept the original values of the patients' sex, age, deprivation, and stage at diagnosis (observed covariate distribution). The aim of these simulations is that the simulated survival times resemble realistically observed survival patterns (see annex).

- Cancer survival times

We designed 2 simulation scenarios: a *simple* one, S1 only containing linear proportional effects of age at diagnosis, and a more *complex* scenario, S2, with non‐proportional and non‐linear effects of age, and non‐proportional effects of categorical stage and deprivation (see Box A).

Survival times for S1 were simulated according to the following function for the log cumulative excess hazard:

$$\ln\left(H_{S1}\left(t;\mathrm{age}\right)\right)=\ln\left(H_{0}\left(t\right)\right)+\beta_{\mathrm{age}}*\mathrm{age}$$

with ln(*H*_0_(*t*)) = *s*(ln(t); γ), *s* being a non‐orthogonalised restricted cubic splines function of ln(t), with up to 3 degrees of freedom, placed at tertiles of the distribution of times.

Survival times for S2 were simulated according to the following function for the log cumulative excess hazard:

$$\ln\left(H_{S2}\left(t;\mathrm{age};\text{stage};\text{deprivation}\right)\right)=\ln\left(H_{0}\left(t\right)\right)+f_{\mathrm{age}}\left(\mathrm{age}\right)*\left(1+\ln\left(t\right)\right)+\sum_{i=2,3,4}\left(\beta_{{\text{stage}}_{i}},*,{\text{stage}}_{i},+,\alpha_{{\text{stage}}_{i}},*,\ln,\left(t\right),*,{\text{stage}}_{i}\right)+\sum_{i=2,3,4,5}\left(\beta_{{\mathrm{dep}}_{i}},*,{\mathrm{dep}}_{i},+,\alpha_{{\mathrm{dep}}_{i}},*,\ln,\left(t\right),*,{\mathrm{dep}}_{i}\right)$$

with ln(*H*_0_(*t*)) = *s*(ln(t); γ), *s* being a non‐orthogonalised, restricted cubic splines function of ln(t) with up to 3 degrees of freedom, placed at tertiles of the distribution of times.

A general algorithm involving numerical integration and root‐finding techniques generated the cancer‐specific survival times from these complex parametric distributions.^18^ We used the survsim command implemented in STATA.^19^

Overall survival time is the minimum between cancer‐specific survival times, as simulated in S1 or S2, expected survival times derived from population life tables and the maximum follow‐up time (8 years). From each simulated dataset, we retained the simulated expected, cancer and overall survival times, and the corresponding vital status indicators.

To make sure our simulated excess hazard and survival curves are realistic, we compared them to the original real‐life hazard and survival curves (Figure 1 in Annex). More details are provided in the tables of bias and coverage (Annex) for both scenarios S1 and S2.

Because the process that generated the survival times is known, it is straightforward to assess the properties of REw calculated in several different estimation models. The estimation models M1 and M2 are well‐specified as they include the same variable structure and form of effects than the simulation scenarios S1 and S2, respectively. The other models M3 to M10 are mis‐specified because simulation and estimation models differ (see Box A).

We expect 1000 simulated datasets to be sufficient to offer a good overview of the properties of REw. All models were fitted on each of the 1000 simulated datasets for S1 and S2, and REw, REw(t), and local REw were calculated and their values retained for the assessment of their properties.

Excess hazard models and cause‐specific hazard models both estimate the same quantity: an estimate of net survival can be derived from both strategies when cause of death is reliably known. Similar agreement is therefore expected between the values of RE measured in cause‐specific and REw in relative survival settings.

### 3.2 REw—Weighting system

**Figure 2:**
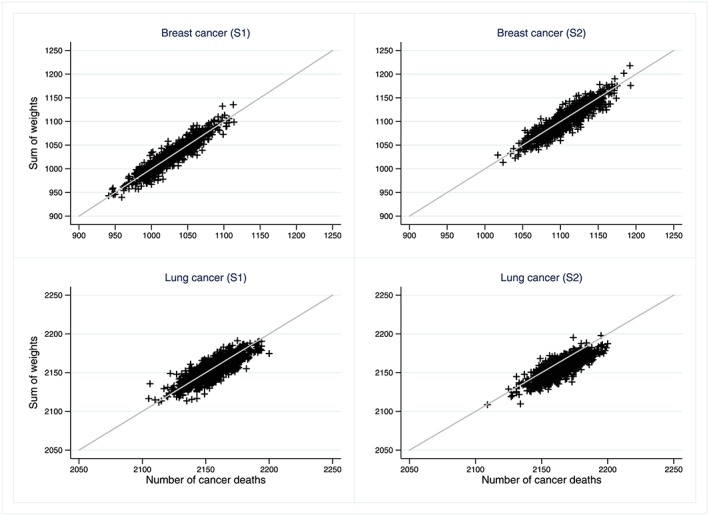
Sum of weights and actual number of cancer deaths, for each of a 1000 simulated datasets, by cancer and simulation scenario. S1: Simulation scenario 1, linear proportional effect of age at diagnosis; S2: Simulation scenario 2, non‐linear non‐proportional effect of age, non‐proportional effects of categorical stage and deprivation [Colour figure can be viewed at http://wileyonlinelibrary.com]

Each individual contribution to REw was weighted by the probability that the event represents a death from cancer. The sum of these weights over all patients who died is an estimate of the number of cancer deaths in the population. Figure 2 compares the actual number of cancer deaths to the sum of weights, for each of the 2 simulation scenarios S1 and S2 for breast and lung cancers.

**Box A. Simulation and estimation scenarios**

**S1**: **Simulation scenario 1, linear proportional effect of age at diagnosis**
M1: Linear proportional effect of age
M3: Linear non‐proportional effect of age
M4: Non‐linear proportional effect of age
M5: Non‐linear non‐proportional effect of age
**S2**: **Simulation scenario 2, non‐linear non‐proportional effect of age, non‐proportional effects of categorical stage and deprivation**
M2: Non‐linear non‐proportional effect of age, non‐proportional effects of categorical stage and deprivation
M6: Non‐linear non‐proportional effect of age, non‐proportional effect of categorical deprivation
M7: Non‐linear non‐proportional effect of age, non‐proportional effect of categorical stage
M8: Linear proportional effect of age, categorical stage and deprivation
M9: Non‐linear non‐proportional effect of age, proportional effect of categorical stage, non‐proportional effect of categorical deprivation
M10: Non‐linear non‐proportional effect of age, non‐proportional effect of categorical stage, proportional effect of categorical deprivation

Over the 8 years of follow‐up, there were on average 1070 breast cancer deaths among the breast cancer patients, ie, 18.4% of patients with breast cancer representing around 60% of deaths; and on average 2159 lung cancer deaths in patients with lung cancer, ie, 90% of patients representing 95% of deaths. Over the 1000 datasets simulated in each of the 2 scenarios, the sum of the weights, used in the calculation of REw, agreed with the actual number of cancer deaths, used in the cause‐specific setting (Figure 2).

**Figure 3:**
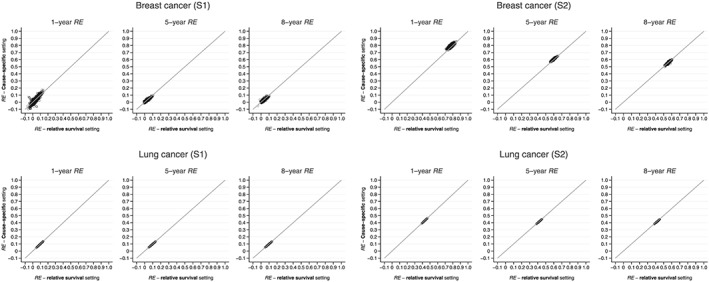
Comparison of RE obtained in cause‐specific and relative survival settings, by cancer and simulation scenario

The agreement between REw values obtained from relative and RE in cause‐specific approaches was nearly perfect, both in simulation scenarios S1 and S2 for breast and lung cancers, and at 1, 5 and 8 years after diagnosis (Figure 3). The larger variability observed in the breast cancer plots was expected and shows the greater instability of the excess hazard models due to the smaller portion that the breast cancer deaths represents among all deaths in that population (60%), contrasting with the burden of lung cancer deaths in lung cancer patients (95%).

We explored a critical scenario in which cancer mortality is very low compared with all‐cause mortality: we selected stage I to II breast cancer patients aged 70 to 99 years at diagnosis. In that sample, REw was still behaving properly despite weights that were slightly over‐estimated. That over‐estimation can have an increasing or decreasing impact on REw depending on the directions of the effects of factors included in both the life table and the excess hazard model.

It is good practice to report the estimated number of cancer deaths, and their proportion among all deaths, as estimated by the sum of weights, so the interpretation of the outputs is given the required caution. Some degree of instability in the estimates of effects is indeed expected in excess hazard models where there is a low proportion of cancer deaths among all deaths.^17^ REw is based on the excess hazard model and therefore suffers twice (through weighting and ranking of events) in such situations. In practice, we follow the recommendation from Sasieni that excess hazard model is best used when the proportion of death due to the disease of interest is between 30% and 90%.^20^

### 3.3 REw—Properties

**Figure 4:**
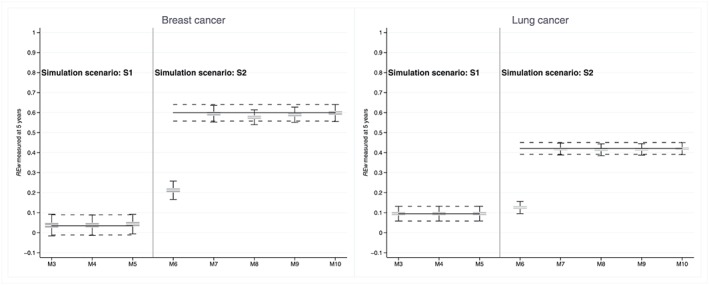
REw measured at 5 years, using different well‐specified (M1, M2, plain lines) and mis‐specified (M3‐M10) models, by cancer and simulation scenario. S1: Simulation scenario 1, linear proportional effect of age at diagnosis; M1: Linear proportional effect of age (plain line across M3‐M5); M3: Linear non‐proportional effect of age; M4: Non‐linear proportional effect of age; M5: Non‐linear non‐proportional effect of age; S2: Simulation scenario 2, non‐linear non‐proportional effect of age, non‐proportional effects of categorical stage and deprivation (plain line across M6‐M10); M2: Non‐linear non‐proportional effect of age, non‐proportional effects of categorical stage and deprivation; M6: Non‐linear non‐proportional effect of age, non‐proportional effect of categorical deprivation; M7: Non‐linear non‐proportional effect of age, non‐proportional effect of categorical stage; M8: Linear proportional effect of age, categorical stage and deprivation; M9: Non‐linear non‐proportional effect of age, proportional effect of categorical stage, non‐proportional effect of categorical deprivation; M10: Non‐linear non‐proportional effect of age, non‐proportional effect of categorical stage, proportional effect of categorical deprivation

Mis‐specifying the form of the effects of the main prognostic factors hardly affected REw. In simulation scenario S1 (simple linear proportional effect of age), the over‐parameterisation of age in the modelling, by inclusion of non‐linear and/or non‐proportional effects of age (models M3‐M5), did not alter REw: median REw, at 0.035 (breast) and 0.095 (lung) with model M1, increased to 0.037 to 0.043 (breast) and remained unchanged for lung (Figure 4).

The impact of stage, a strong predictor of survival, on REw is obvious when stage was omitted in the modelling (M6) while it was present in the simulation scenario (S2): median REw decreased from 0.600 (M2) to 0.213 (M6) for breast, and from 0.421 (M2) to 0.126 (M6) for lung (Figure 4). All other types of model mis‐specification, such as omitting deprivation (M7), or omitting/including non‐linearity or non‐proportionality of age, deprivation or stage (M8‐M10), did not have any strong impact on REw: for both breast and lung cancers, the largest differences in median REw occurred with under‐parameterisation of stage, ie, lack of non‐proportionality of the effect (M8, M9), and still showed a difference in median REw as small as 0.02 or less.

REw is robust to model mis‐specification because the ranking of the individual hazards is unaffected by estimated changes in the strength of the effects only. M6, in which the effect of stage is ignored, shows greater impact on REw due to large changes in the ranking of observations.

The local REw was calculated using 20 events around each index event. This choice resulted in windows of varying lengths: stable at around 25 days all through the follow‐up for breast cancer patients, whereas it started at less than 20 days for the first year of lung cancer follow‐up, and then gradually increased to 450 days beyond 7 years. Indeed, over 75% of deaths occurred in the year following the lung cancer diagnosis, although it takes 5 years to observe 75% of deaths for breast cancer patients.

**Figure 5:**
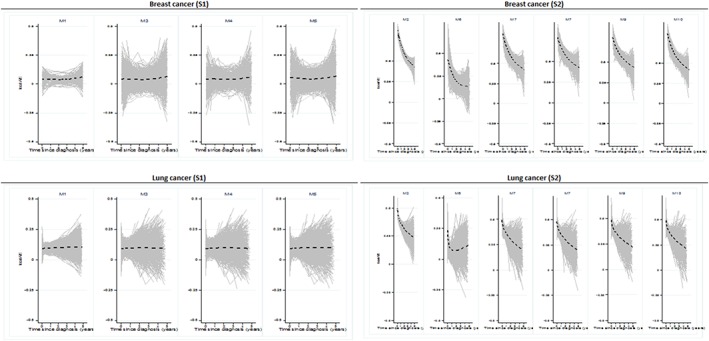
Local REw measured up to 5 years, for different well‐specified (M1, M2) and mis‐specified models (M3‐M5 and M6‐M10): breast and lung cancers. S1: Simulation scenario 1, linear proportional effect of age at diagnosis; M1: Linear proportional effect of age; M3: Linear non‐proportional effect of age; M4: Non‐linear proportional effect of age; M5: Non‐linear non‐proportional effect of age; S2: Simulation scenario 2, non‐linear non‐proportional effect of age, non‐proportional effects of categorical stage and deprivation; M2: Non‐linear non‐proportional effect of age, non‐proportional effects of categorical stage and deprivation; M6: Non‐linear non‐proportional effect of age, non‐proportional effect of categorical deprivation; M7: Non‐linear non‐proportional effect of age, non‐proportional effect of categorical stage; M8: Linear proportional effect of age, categorical stage and deprivation; M9: Non‐linear non‐proportional effect of age, proportional effect of categorical stage, non‐proportional effect of categorical deprivation; M10: Non‐linear non‐proportional effect of age, non‐proportional effect of categorical stage, proportional effect of categorical deprivation

There was little variation between the 1000 local REw curves when simulation and estimation models coincided, ie, M1 in S1 and M2 in S2 (Figure 5). The general patterns of local REw seen in well‐specified models were however preserved for mis‐specified estimation models. In the simple scenario S1, local REw remained relatively constant with time since diagnosis for all models. For the more complex simulation scenario S2, the local REw curves decreased with time for all models. We further explored that decrease in local REw to understand what effect it reflected. We looked at simulated data following 2 additional scenarios, S3 and S4: S3 included linear proportional effects of age, and proportional effects of categorical deprivation and stage at diagnosis, while S4 included non‐linear and non‐proportional effects of age, and non‐proportional effects of categorical deprivation. While the local REw curves also decreased in simulation scenario S4, they remained constant in S3 indicating that non‐proportional effects of age and other factors, rather than the adjustment for stage, triggered a decreasing local REw in S2.

The weighting initially proposed in Stare et al takes good account of random censoring through time. However, in the event that some cohorts of patients are all censored at a fixed date, such as due to administrative censoring when performing a complete study design, this weighting was not sufficient to cope for such large amount of censored information, often tied. We advise to break ties by simply adding or subtracting a small fraction of time to the survival times that tie. It will then prevent the spurious increasing REw emanating from large proportions of patients censored at similar times after diagnosis. Without this correction, local and overall REw will also converge to 1 from the time heavy censoring starts occurring. For users analysing a cohort study design, it is advised to measure the cumulative REw right before the administrative censoring happens.

## 4 APPLICATION: COMPLEX MULTIVARIABLE MODELLING

Given the availability of potential predictors of cancer survival in England, we selected patients diagnosed with colon cancer in 2011 to 2013 (*n* = 9300) or non‐small cell lung cancer in 2012 (*n* = 5958), with follow‐up until the end of 2014. We selected a 25% random sample of patients with valid information on sex, age at diagnosis, deprivation, stage at diagnosis, major surgical treatment, and comorbidity (Charlson index, CCI) for all patients and additional information on performance status and route to diagnosis for lung cancer patients only (Table 1).

**Table 1:** Number and proportion of patients, by stage at diagnosis and each of the main explanatory factors: Lung cancer patients diagnosed in 2012, colon cancer patients diagnosed in 2011 to 2013 in England

	Non‐Small Cell Lung Cancer																			
	Men										Women									
	Stage I		Stage II		Stage III		Stage IV		Total		Stage I		Stage II		Stage III		Stage IV		Total	
Age at diagnosis																				
Mean (sd)	72.8	(10)	72.9	(10.2)	72.1	(10.06)	72.3	(10.4)			72.6	(10.7)	72.6	(11)	72.1	(10.5)	72.2	(11.1)		
Treatment	No.	%	No.	%	No.	%	No.	%	No.	%	No.	%	No.	%	No.	%	No.	%	No.	%
No major surgical treatment	223	44.2	149	52.3	753	89.1	1658	99.0	2783	84.1	214	44.7	123	52.1	528	89.0	1327	98.9	2192	82.7
Major surgical treatment	281	55.8	136	47.7	92	10.9	16	1.0	525	15.9	265	55.3	113	47.9	65	11.0	15	1.1	458	17.3
Emergency presentation																				
No	425	84.3	237	83.2	657	77.8	1016	60.7	2335	70.6	367	76.6	186	78.8	452	76.2	767	57.2	1772	66.9
Yes	79	15.7	48	16.8	188	22.2	658	39.3	973	29.4	112	23.4	50	21.2	141	23.8	575	42.8	878	33.1
Performance status																				
High—0	168	33.3	87	30.5	201	23.8	230	13.7	686	20.7	138	28.8	66	28.0	122	20.6	177	13.2	503	19.0
1	186	36.9	114	40.0	321	38.0	521	31.1	1142	34.5	171	35.7	86	36.4	220	37.1	435	32.4	912	34.4
2	83	16.5	47	16.5	147	17.4	388	23.2	665	20.1	77	16.1	48	20.3	108	18.2	268	20.0	501	18.9
3	51	10.1	27	9.5	121	14.3	370	22.1	569	17.2	63	13.2	29	12.3	115	19.4	306	22.8	513	19.4
4	7	1.4	3	1.1	36	4.3	126	7.5	172	5.2	12	2.5	3	1.3	20	3.4	124	9.2	159	6.0
Low—5	9	1.8	7	2.5	19	2.2	39	2.3	74	2.2	18	3.8	4	1.7	8	1.3	32	2.4	62	2.3
Deprivation quintile																				
Least deprived	81	16.1	35	12.3	107	12.7	241	14.4	464	14.0	54	11.3	32	13.6	74	12.5	165	12.3	325	12.3
2	84	16.7	51	17.9	139	16.4	269	16.1	543	16.4	85	17.7	39	16.5	93	15.7	194	14.5	411	15.5
3	93	18.5	56	19.6	161	19.1	355	21.2	665	20.1	83	17.3	50	21.2	130	21.9	264	19.7	527	19.9
4	100	19.8	70	24.6	224	26.5	365	21.8	759	22.9	119	24.8	50	21.2	141	23.8	367	27.3	677	25.5
Most deprived	146	29.0	73	25.6	214	25.3	444	26.5	877	26.5	138	28.8	65	27.5	155	26.1	352	26.2	710	26.8
Charlson comorbidity score																				
None—0	256	50.8	165	57.9	524	62.0	1054	63.0	1999	60.4	251	52.4	136	57.6	373	62.9	917	68.3	1677	63.3
1	113	22.4	52	18.2	141	16.7	295	17.6	601	18.2	127	26.5	55	23.3	107	18.0	218	16.2	507	19.1
2	54	10.7	37	13.0	83	9.8	169	10.1	343	10.4	47	9.8	28	11.9	56	9.4	98	7.3	229	8.6
>2	81	16.1	31	11.0	97	11.3	156	9.4	365	11.0	54	11.2	17	7.2	57	9.6	109	7.9	237	8.9
Total	504	100.0	285	100.0	845	100.0	1674	100.0	3308	100.0	479	100.0	236	100.0	593	100.0	1342	100.0	2650	100.0

	Colon cancer																			
	Men										Women									
	Stage I		Stage II		Stage III		Stage IV		Total		Stage I		Stage II		Stage III		Stage IV		Total	
Age at diagnosis																				
Mean (sd)	70.9	(11.2)	72.5	(11.6)	69.8	(12.6)	72.0	(12)			70.5	(13.3)	73.8	(12)	72.1	(12.7)	71.9	(13.7)		
Treatment	No.	%	No.	%	No.	%	No.	%	No.	%	No.	%	No.	%	No.	%	No.	%	No.	%
No major surgical treatment	31	4.7	41	2.8	59	4.5	393	25.4	524	10.6	27	4.9	49	3.7	68	6.0	404	30.1	548	12.6
Major emergency surgery	33	5.0	264	18.2	268	20.6	258	16.7	823	16.6	35	6.3	258	19.7	283	24.8	241	17.9	817	18.8
Major elective surgery	421	64.4	1028	71.0	845	64.9	321	20.8	2615	52.8	377	67.8	914	69.7	680	59.7	256	19.1	2227	51.2
Minor surgery	169	25.8	115	7.9	131	10.1	573	37.1	988	20.0	117	21.0	91	6.9	108	9.5	442	32.9	758	17.4
Deprivation quintile																				
Least deprived	140	21.4	318	22.0	290	22.3	331	21.4	1079	21.8	131	23.6	304	23.2	291	25.5	262	19.5	988	22.7
2	154	23.5	325	22.4	307	23.6	343	22.2	1129	22.8	118	21.2	290	22.1	247	21.7	293	21.8	948	21.8
3	150	22.9	318	22.0	266	20.4	329	21.3	1063	21.5	121	21.8	270	20.6	215	18.9	280	20.8	886	20.4
4	117	17.9	269	18.6	251	19.3	321	20.8	958	19.4	114	20.5	238	18.1	205	18.0	301	22.4	858	19.7
Most deprived	93	14.2	218	15.1	189	14.5	221	14.3	721	14.6	72	12.9	210	16.0	181	15.9	207	15.4	670	15.4
Charlson comorbidity score																				
None ‐ 0	477	72.9	1061	73.3	1015	77.9	1146	74.2	3699	74.7	440	79.1	1037	79.0	923	81.0	1076	80.1	3476	79.9
1	78	11.9	194	13.4	122	9.4	179	11.6	573	11.6	65	11.7	142	10.8	106	9.3	139	10.3	452	10.4
2	49	7.5	108	7.5	91	7.0	116	7.5	364	7.4	31	5.6	78	5.9	62	5.4	66	4.9	237	5.4
>2	50	7.7	85	5.8	75	5.8	104	6.8	314	6.3	20	3.7	55	4.3	48	4.3	62	4.6	185	4.3
Total	654	100.0	1448	100.0	1303	100.0	1545	100.0	4950	100.0	556	100.0	1312	100.0	1139	100.0	1343	100.0	4350	100.0

The initial parametric log‐cumulative excess hazard models, stratified by sex, included age at diagnosis and deprivation, and expected hazards were provided by life tables defined by sex, single year of age, and deprivation. We aimed to measure the explained variation of the increasingly more complex models to reflect the explained variation of each factor successively added into the models.

The sum of the weights derived for the calculation of REw quantified the proportion of cancer deaths among all deaths. Of the 40.6% (42.0%) colon cancer patients dying through the follow‐up, we estimated that 79.0% (83.4%) died of cancer in men (women); and of the 86.4% (81.3%) of dead men (women) lung cancer patients, 94.3% (95.0%) died of their cancer.

Table 2 shows that REw reached 0.22 (95%CI: 0.16–0.28) (REw = 0.26, 95%CI: 0.20–0.31) in men (women) with colon cancer, and 0.14 (95%CI: 0.11–0.17) (REw = 0.16, 95%CI: 0.12–0.19) in men (women) with lung cancer at 12 months after diagnosis, for models adjusted for age and deprivation only, ie, the baseline model. Full adjustment for all available covariables increased REw to 0.69 (95% CI: 0.67–0.70) (REw = 0.67, 95%CI: 0.66–0.69) in men (women) with colon cancer and 0.56 (95%CI: 0.54–0.58) (REw = 0.58, 95%CI: 0.56–0.60) in men (women) with lung cancer. Stage accounted for most of the increase in colon cancer, explaining 61.8% (53.5%) in men (women) of the explained variation of the full model, and increasing the baseline REw by over 150%. In lung cancer, performance status and stage showed the largest increase in REw, from the minimum initial model: around 200%, with an absolute change in REw of 0.29 (0.30) and 0.28 (0.29) in men (women) respectively; but in a full model, their respective shares represented 12.4% (10.8%) and 10.5% (7.4%) in men (women), suggesting correlation between variables such as treatment and stage, or emergency presentation and stage.

**Table 2:** Multivariable model: Explained variation (*REw*) measured at 12 months after diagnosis, for overall models and individual variables

				Change^a^ in REw			
				Inclusion^b^		Exclusion^b^	
		REw at 12 months (95% CI)		Difference in REw	Proportion of Initial Model (%)	Difference in REw	Proportion of Full Model (%)
Colon cancer	
Men							
Initial model:	*Age, deprivation*	*0.221*	*(0.160; 0.282)*				
	Age, deprivation, stage	0.671	(0.657; 0.686)	0.450	203.5	0.427	61.8
	Age, deprivation, treatment^c^	0.251	(0.195; 0.307)	0.030	13.5	0.016	2.4
	Age, deprivation, Charlson Comorbidty index (CCI)	0.232	(0.171; 0.292)	0.010	4.7	0.003	0.4
Full model:	*Age, deprivation, stage, treatment, CCI*	*0.690*	*(0.676; 0.704)*				
Women	
Initial model:	*Age, deprivation*	*0.256*	*(0.198; 0.314)*				
	Age, deprivation, stage	0.660	(0.644; 0.675)	0.403	157.5	0.359	53.5
	Age, deprivation, treatment^c^	0.290	(0.241; 0.340)	0.034	13.4	0.010	1.4
	Age, deprivation, Charlson Comorbidity index (CCI)	0.271	(0.214; 0.329)	0.015	6.0	0.002	0.3
Full model:	*Age, deprivation, stage, treatment, CCI*	*0.671*	*(0.656; 0.686)*				
Non‐small cell lung cancer							
Men							
Initial model:	*Age, deprivation*	*0.141*	*(0.112; 0.171)*				
	Age, deprivation, stage	0.422	(0.403; 0.441)	0.280	198.5	0.058	10.5
	Age, deprivation, treatment^d^	0.257	(0.235; 0.280)	0.116	81.9	0.003	0.6
	Age, deprivation, Charlson Comorbidity index (CCI)	0.141	(0.111; 0.170)	−0.001	−0.5	0.000	0.1
	Age, deprivation, performance status (PS)	0.434	(0.409; 0.459)	0.293	207.3	0.069	12.4
	Age, deprivation, presentation (EP vs non‐EP)	0.325	(0.295; 0.354)	0.183	129.8	0.013	2.4
Full model:	*Age, deprivation, stage,treatment, CCI, PS, presentation*	*0.558*	*(0.539; 0.576)*				
Women							
Initial model:	*Age, deprivation*	*0.155*	*(0.120; 0.191)*				
	Age, deprivation, stage	0.442	(0.421; 0.463)	0.287	185.1	0.043	7.4
	Age, deprivation, treatment^d^	0.299	(0.274; 0.324)	0.144	92.8	0.006	1.0
	Age, deprivation, Charlson Comorbidity index (CCI)	0.157	(0.121; 0.192)	0.002	1.0	−0.001	−0.2
	Age, deprivation, performance status (PS)	0.455	(0.427; 0.484)	0.300	193.6	0.063	10.8
	Age, deprivation, presentation (EP vs non‐EP)	0.352	(0.318; 0.387)	0.197	127.2	0.020	3.4
Full model:	*Age, deprivation, stage, treatment, CCI, PS, presentation*	*0.584*	*(0.564;0.604)*				

Modelled effects: Age: non‐linear and non‐proportional, Deprivation: categorical, non‐proportional, Stage: categorical, non‐proportional, Treatment: categorical, non‐proportional, CCI: linear, non‐proportional, Performance status: categorical, Presentation binary: emergency presentation (EP) versus non‐emergency.

a Change is measured as the arithmetic difference between the initial (inclusion) or full (exclusion) model REw and the model that includes the specific variable. That difference is expressed as a proportion of the initial (inclusion) or full (exclusion) model.

b Inclusion: change in REw with the addition of the index variable to a model including age and deprivation. Exclusion: change in Rew with the removal of the index variable from the full model.

c The variable “treatment” represents major surgical resection.

d The variable “treatment” represents both treatment and the route to diagnosis: 1—no treatment, 2—emergency major surgery, 3—elective major surgery, 4—minor surgery.

**Figure 6:**
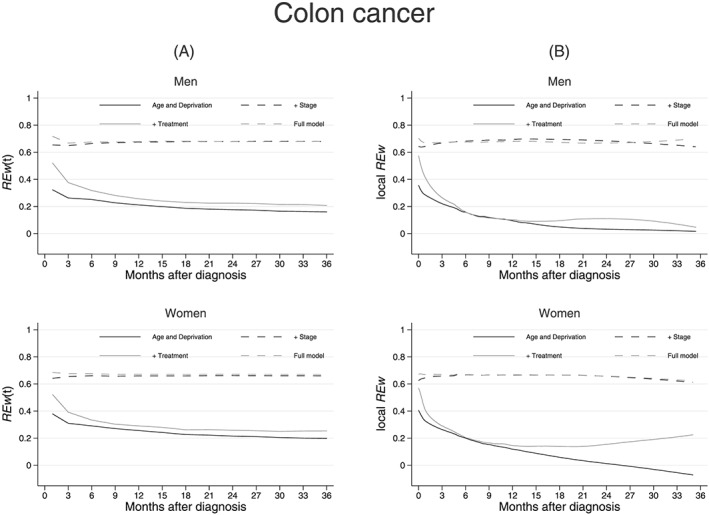
Multivariable models: (A) explained variation measured at 1 month and every 3 months after diagnosis, (B) smoothed local RE up to 3 years after diagnosis, for models adjusted for the effects of age and deprivation, and stage, and treatment. Colon cancer patients diagnosed in 2011 to 2013, selected for their valid stage at diagnosis: 4950 men and 4350 women the curve for comorbidity is not presented here as it is undistinguishable from the age and deprivation model Notes: (1) RE(t) and local RE can have values between −1 and +1 (2) Cumulative RE, RE(t) is calculated at month 1, 3, 6…36 after diagnosis (3) Local RE is calculated using information from 10 events on either side of the index event. The smoothed (lowess with mean smoother) curve is presented here

**Figure 7:**
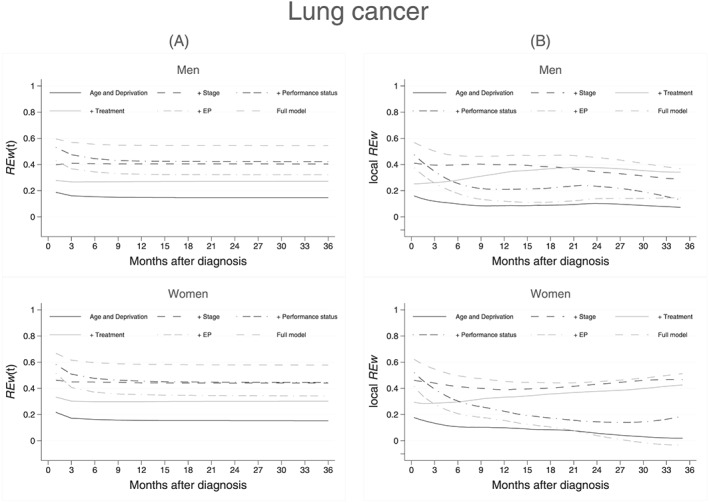
Multivariable models: (A) explained variation measured at 1 month and every 3 months after diagnosis, (B) smoothed local RE up to 3 years after diagnosis, for models adjusted for the effects of age and deprivation, and stage, treatment, performance status, and emergency presentation. Non‐small cell lung cancer patients diagnosed in 2012, selected for their valid stage and performance status at diagnosis: 3308 men and 2650 women. The curve for comorbidity is not presented here as it is undistinguishable from the age and deprivation model Notes: (1) RE(t) and local RE can have values between −1 and +1 (2) Cumulative RE, RE(t) is calculated at month 1, 3, 6…36 after diagnosis (3) Local RE is calculated using information from 10 events on either side of the index event. The smoothed (lowess with mean smoother) curve is presented here

We then measured time‐varying REw at 1 month and every 3 months following diagnosis, up to 3 years (Figures 6A and 7A). In colon cancer patients, there is a clear distinction between models that do and do not contain stage at diagnosis. In models excluding stage, REw(t) was stable from 12 months after a sharp decrease in the first 6 months and then slight decrease until the 12^th^ month (Figure 6A). The local REw showed evidence that at 2 years after diagnosis, models that contained the surgical treatment variable, without stage at diagnosis, displayed an increased local REw (Figure 6B).

In lung cancer models, the time‐varying REw increased from the baseline age and deprivation model with any additional variable: REw(t) was stable after a slight decrease until 3 months, mostly in women, and in models adjusted for emergency presentation (from over 0.5 in women to less than 0.4, Figure 7A). Additionally, the curves reflecting presence of stage and performance status reflect perfectly the large contribution of performance status at the start of the follow‐up, and the constant contribution of stage. The patterns of the local REw curves are suggestive of a late treatment effect: generally decreasing over time for models including emergency presentation or performance status but increasing for the model including surgical treatment information (Figure 7B).

By definition of the local REw, the shapes of the smoothed curves are only slightly influenced by the number of events included in the windows around each index event. For both colon and lung cancers, including 10 events on either side of the index event resulted in windows of times varying between a day and 50 days for lung cancer or between a day and over 75 days for colon cancer in the 3 years of follow‐up. The degree of smoothing will also likely impact the shape of the local REw curves. Furthermore, the cumulative nature of the overall and time‐varying REw means that they are likely impacted by the high proportions of death happening at the beginning of the follow up: 50% of all deaths occurred by the 3^rd^ and 9^th^ months of follow‐up in lung and colon cancer, respectively, explaining why REw(t) was mostly flat beyond these times.

## 5 DISCUSSION

We presented here an adaptation of the RE measure for event history data to excess hazard modelling. We offer a new tool to quantify the variation in disease‐specific outcome explained by the available predictive factors. In this context, REw can be measured at given time points following diagnosis and plotted as a function of time. Additional exploratory insight is provided by a “local REw”, calculated using a window of events around each event time. That function of time can be very unstable, and the smoothed curve is useful to look at the general trend in the variation in RE by the model. Although dependent on death patterns, these time‐varying versions of REw help understand better when specific factors have strongest impact on survival.

The differences between local REw and REw(t) curves can be seen similarly to the differences between hazard and cumulative hazard curves. The cumulative hazard curve is a cumulative measure, whereby hardly affected by local effects seen in the instantaneous hazard curve. REw(t) is the cumulative REw, heavily impacted by the first few months following the diagnosis, where most cancer‐related deaths occur. If one is interested in changes in explained variation due to, say late treatment effects or changes in the composition of the cohort of patients (younger ages, fewer late stage patients, fitter patients…), the local REw will provide such information. Furthermore, in the context of dynamic data and dynamic models, local REw will be providing the necessary time‐varying measure of explained variation.

Furthermore, local REw and REw(t) are informative for comparison between studies, or when varying follow‐up times are available, because the overall measure REw will vary with the available follow‐up.^21^

Further research in the number of events to include in the calculation of the local REw show very little variation in the smoothed functions. Only the heights of the spikes seen in the un‐smoothed local REw curves are affected, and hence the tail of the smoothed curve, where the number of events is more scarce. We advise researchers to use 20 events, as a default size, and depending on the cancer lethality, check for the impact of using much smaller (say 4–10 events) or much larger (30–40 events) number of events. The local REw curve will, to some extent, depend on the number of events as well as the amount of smoothing applied.

The weighting system proposed here for the relative survival setting keeps the simplicity and the intuition of the original RE used in the overall and cause‐specific settings. It also retains the original RE measure's properties such as model‐free interpretation. Furthermore, the weighted measure REw in the relative survival setting is equivalent to RE in the cause‐specific setting.

Multidimensional models defined on the log or log‐cumulative hazard scales can now be routinely used to estimate excess hazard from cancer.^3^, ^11^ These models often include complex non‐linear and non‐proportional effects of a variety of factors that may impact levels of survival. Therefore, the regression coefficients are not straightforward to interpret, and strong predictors are often hard to pin down. We propose to look at differences in REw between models to quantify the proportion of variation explained by a given factor. Our illustration shows that for lung cancer patients, performance status explained the largest amount of variation in survival between patients, particularly in the early months following diagnosis. Performance status, although well‐known and discussed in Multi‐Disciplinary Team meetings, is rarely accounted for in epidemiology, mainly because of its unavailability in the routine cancer registration datasets. Such high explanatory power for that variable could trigger its availability at least in specialised cancer registry datasets.

Low proportions of explained variation for single covariable in the full model, whereas each additional variable adds, individually, a lot to the explained variation of the baseline model, indicate high correlation between factors. It could reflect a high adherence to guidelines such that whole groups of patients got administered the same treatment, or were diagnosed via a given ideal route.

Despite the measure being dependent on the excess hazard model through the weighting and through the ranking of observations, REw proved a great stability to model specification. REw was largely insensitive to over‐parameterisation or under‐parameterisation of the variables used in the simulation model. Non‐linear or non‐proportional effects, although they may reflect better the reality of the estimated disease‐specific survival, will not impact dramatically the order at which patients will experience the event of interest.

REw, like RE, is not exact. Small sample sizes or low number of deaths due to the disease of interest will increase variability around the estimated REw. Therefore, we advise users to report the variance or confidence interval obtained around the estimated REw. Similar to RE, REw estimates may be biased for a factor with a small effect.^13^ However, the bias will become negligible as the sample size increases.

Further developments will include testing the REw on dynamic models that include time‐varying variables,^22^ and in hierarchical models.^2^ The variation explained by these models may be greater, because they allow the effect of time‐varying variables to be modelled and, hence, measures of prognostic factors that are updated over time since the cancer diagnosis.

## Supporting information

Data S1 Supporting Information

## References

[1] Lambert PC , Royston P . Further development of flexible parametric models for survival analysis. The Stata Journal. 2009;9:265‐290.

[2] Charvat H , Remontet L , Bossard N , et al. A multilevel excess hazard model to estimate net survival on hierarchical data allowing for non‐linear and non‐proportional effects of covariates. Stat Med. 2016;35(18):3066‐3084. 26924122 10.1002/sim.6881

[3] Remontet L , Bossard N , Belot A , et al. An overall strategy based on regression models to estimate relative survival and model the effects of prognostic factors in cancer survival studies. Stat Med. 2007;26(10):2214‐2228. 16900570 10.1002/sim.2656

[4] Giorgi R , Abrahamowicz M , Quantin C , et al. A relative survival regression model using B‐spline functions to model non‐proportional hazards. Stat Med. 2003;22(17):2767‐2784. 12939785 10.1002/sim.1484

[5] Graf E , Schmoor C , Sauerbrei W , Schumacher M . Assessment and comparison of prognostic classification schemes for survival data. Stat Med. 1999;18(17–18):2529‐2545. 10474158 10.1002/(sici)1097-0258(19990915/30)18:17/18<2529::aid-sim274>3.0.co;2-5

[6] Heagerty PJ , Zheng Y . Survival model predictive accuracy and ROC curves. Biometrics. 2005;61(1):92‐105. 15737082 10.1111/j.0006-341X.2005.030814.x

[7] Pencina MJ , D'Agostino RB . Overall C as a measure of discrimination in survival analysis: model specific population value and confidence interval estimation. Stat Med. 2004;23(13):2109‐2123. 15211606 10.1002/sim.1802

[8] Schemper M , Stare J . Explained variation in survival analysis. Stat Med. 1996;15(19):1999‐2012. 8896135 10.1002/(SICI)1097-0258(19961015)15:19<1999::AID-SIM353>3.0.CO;2-D

[9] Cox DR . Regression models and life‐tables. J R Stat Soc B Methodol. 1972;34(2):187‐220.

[10] Esteve J , Benhamou E , Croasdale M , Raymond L . Relative survival and the estimation of net survival: elements for further discussion. Stat Med. 1990;9(5):529‐538. 2349404 10.1002/sim.4780090506

[11] Nelson CP , Lambert PC , Squire IB , Jones DR . Flexible parametric models for relative survival, with application in coronary heart disease. Stat Med. 2007;26(30):5486‐5498. 17893893 10.1002/sim.3064

[12] Danieli C , Remontet L , Bossard N , et al. Estimating net survival: the importance of allowing for informative censoring. Stat Med. 2012;31(8):775‐786. 22281942 10.1002/sim.4464

[13] Stare J , Perme MP , Henderson R . A measure of explained variation for event history data. Biometrics. 2011;67(3):750‐759. 21155749 10.1111/j.1541-0420.2010.01526.x

[14] Harrell FE Jr , Califf RM , Pryor DB , et al. Evaluating the yield of medical tests. JAMA. 1982;247(18):2543‐2546. 7069920

[15] Schemper M , Henderson R . Predictive accuracy and explained variation in Cox regression. Biometrics. 2000;56(1):249‐255. 10783803 10.1111/j.0006-341x.2000.00249.x

[16] Pohar Perme M , Stare J , Esteve J . On estimation in relative survival. Biometrics. 2012;68(1):113‐120. 21689081 10.1111/j.1541-0420.2011.01640.x

[17] Pohar Perme M , Henderson R , Stare J . An approach to estimation in relative survival regression. Biostatistics. 2009;10(1):136‐146. 18599516 10.1093/biostatistics/kxn021

[18] Crowther MJ , Lambert PC . Simulating biologically plausible complex survival data. Stat Med. 2013;32(23):4118‐4134. 23613458 10.1002/sim.5823

[19] Crowther MJ , Lambert PC . Simulating complex survival data. The Stata Journal. 2012;12(4):674‐687.

[20] Sasieni PD . Proportional excess hazard. Biometrika. 1996;83(1):127‐141.

[21] Kejzar N , Maucort‐Boulch D , Stare J . A note on bias of measures of explained variation for survival data. Stat Med. 2016;35(6):877‐882. 26428056 10.1002/sim.6749

[22] Mauguen A , Rachet B , Mathoulin‐Pelissier S , et al. Dynamic prediction of risk of death using history of cancer recurrences in joint frailty models. Stat Med. 2013;32(30):5366‐5380. 24030899 10.1002/sim.5980

