# Supplementary material for: Explained variation of excess hazard models
Source: Stat Med. 2018 Apr 6;37(14):2284–300. doi: 10.1002/sim.7645 (PMC6001643; doi:10.1002/sim.7645)
Supplement: Supplementary file 1 — Data S1 Supporting Information [file SIM-37-2284-s001.pdf]

Simulation strategy

1. Expected survival time

a. Merge the life table file to the patient file, on sex, deprivation, age and month of diagnosis. Age corresponds to the exact age at diagnosis ($agediag$), truncated at the nearest month (age_m) such that

$$age_m \leq agediag < age_{m+1}$$

$rate_0$ is the population mortality rate merged to the patients at the start of the month of their diagnosis.

Merge again the life table file to the patient file, on sex, deprivation, age at diagnosis + 1 month and the month following their diagnosis.
$rate_1$ is the population mortality rate merged to the patients at the start of the month following their diagnosis.

Linear interpolation between $rate_0$ and $rate_1$ to get the mortality rate that refers exactly to the day of diagnosis: $rate$.
$rate$ is a yearly mortality rate, transform it to a monthly rate.

b. Generate a survival time t_1, following an exponential distribution with $\lambda_1 = rate$:

$$t_1 = -\frac{1}{\lambda_1} * \ln(u_1)\,, u_1 \sim U[0,1]$$

c. Compare t_1 to the length of a month (1):
   - If $t_1 < 1$, patient's expected survival time is t_1. STOP
   - If not, patient's expected survival time is at 1 month, and we proceed to d.

d. Merge the life tables again to the patient file, on sex, deprivation, age at diagnosis +1 month and month of diagnosis +1 month >> $rate_0$
Merge the life tables again to the patient file, on sex, deprivation, age at diagnosis +2 months and month of diagnosis +2 month >> $rate_1$
Linear interpolation between $rate_0$ and $rate_1$ to get the mortality rate that refers exactly to the day of diagnosis: $rate$.
$rate$ is a yearly mortality rate, transform it to a monthly rate.

e. Generate a survival time t_2, following an exponential distribution with $\lambda_2 = rate$:

$$t_2 = -\frac{1}{\lambda_2} * \ln(u_2)$$

f. Compare t_2 to 1:
   - If $t_2 < 1$, patient's expected survival time is $t_2 + 1$ month. STOP
   - If not, patient's expected survival time is at least 2 months, and we proceed to g.

g. Repeat d-f until all patients have an expected survival time, t_{exp} or survive beyond the maximum follow up time.

In summary,

$$t_{exp} = \sum_i \min\left(-\frac{1}{\lambda_i} * \ln(u_i)\,, 1\right) |_{-\frac{1}{\lambda_{i-1}} * \ln(u_{i-1}) > 1}$$

With λ_i the mortality rate of the anniversary day of diagnosis of the ith month following diagnosis.

2. Cancer survival time

a. Parameters of the simulations

We run an excess hazard model, fitted on the log cumulative excess hazard scale. Life tables of population background mortality are merged to the data on sex, deprivation, age and exact time of death through linear interpolation between

- population mortality rate available on the 1st of the month of death at the age at death truncated to the nearest month,
- and the population mortality rate of the 1st of the month that follows the month of death at the age at death + 1 month.

We use the estimated coefficients and hazard function from the excess hazard model to feed into the simulation of a cancer survival time, t_{cancer}:

b. simulations

If we consider the following non-proportional hazards model, expressed in the log cumulative hazard scale:

$$ln\{H_i(t|\boldsymbol{x_i})\} = s\{\ln(t)|\gamma, \boldsymbol{k_0}\} + \sum_{j=1}^{D} \ln(t) * x_{ij} + \boldsymbol{x_i}\beta$$

$\boldsymbol{k_0}$ is a set of knots for the baseline hazard.

The survival function is defined by,

$$S(t|\boldsymbol{x_i}) = \exp\{-\exp(ln\{H_i(t|\boldsymbol{x_i})\})\}$$

And then if t_{cancer} is the simulated survival time, $F(t_{cancer}|\boldsymbol{x_i}) = 1 - S(t_{cancer}|\boldsymbol{x_i}) = u$, we have

$$t_{cancer} = H^{-1}[-\ln(u)]$$

In the complex situation in which we are, *H* cannot be directly inverted so numerical integration (to obtain a closed form for *H*) and iterative root finding are used (to solve the equation for the simulated time).

[ref: Lambert and Royston, STATA journal 9, 2009 and Crowther and Lambert, Stats in Med 2013]

3. Overall survival time

A patient's survival time *t* is then taken as the minimum between the expected survival time and the cancer survival time.

$$t = \min(t_{exp}, t_{cancer})$$

Analysis strategy

To bring in information on population mortality at the time of death, we merge life tables to the data on sex, deprivation, age truncated to the nearest year and year at time of death. The mortality rate is measured on the 1st of July of that given year (linear interpolation between the mortality rates measured on the 1st of January of the index year and the following year). The general form of the model on the log cumulative excess hazard scale, including potential time-dependent effects is:

$$\begin{aligned} ln\{H_i(t|\boldsymbol{x_i})\} &= ln\{H_{i,obs}(t|\boldsymbol{x_i}) - H_{i,exp}(1st\,Jul|age, year, deprivation, sex)\} \\ &= s\{\ln(t)|\gamma, \boldsymbol{k_0}\} + \sum_{j=1}^{D} \ln(t) * x_{ij} + \boldsymbol{x_i}\beta \end{aligned}$$

Annex: Table 1: Summary of the simulations

Model		Time-fixed effects									Time-varying effects								
**Breast**		**Linear age**	**Non-linear age**	**Stage1**	**Stage 2**	**Stage 3**	**Dep. 2**	**Dep. 3**	**Dep. 4**	**Dep. 5**	**Linear age**	**Non-linear age**	**Stage1**	**Stage 2**	**Stage 3**	**Dep. 2**	**Dep. 3**	**Dep. 4**	**Dep. 5**
**S1**	**original coeff**	0.0042																	
	**bias**	0.0004																	
	**relative bias**	0.0922																	
	**coverage**	97																	
**S2**	**original coeff**	0.2519	-0.1764	-5.4687	-3.1933	-1.5921	0.2955	0.3264	0.0863	0.3225	-0.0880	0.0416	1.0362	0.6856	0.4513	-0.1125	-0.1010	-0.0392	-0.0797
	**bias**	0.0000	-0.0090	0.5705	-0.1105	-0.0780	-0.0328	0.0190	0.0302	0.0554	0.0029	0.0018	-0.2140	0.0624	0.0425	0.0035	-0.0331	-0.0538	-0.0125
	**relative bias**	0.0002	0.0512	-0.1043	0.0346	0.0490	-0.1109	0.0583	0.3495	0.1718	-0.0326	0.0431	-0.2065	0.0910	0.0942	-0.0311	0.3275	1.3722	0.1570
	**coverage**	95.6	96	72.8	94.2	94	95.9	96.2	95.6	94.3	95.7	95.8	81.1	92.3	93.2	95.4	95.4	92.2	95.7
**Lung**																			
**S1**	**original coeff**	0.0173																	
	**bias**	0.0000																	
	**relative bias**	0.0027																	
	**coverage**	95.5																	
**S2**	**original coeff**	0.1970	-0.0140	-2.2191	-1.6769	-0.7140	0.0974	0.0966	0.0476	0.1576	-0.0497	0.0347	0.4037	0.3509	0.2602	0.0426	0.0766	-0.0111	0.0285
	**bias**	0.0012	-0.0011	-0.0289	-0.0385	0.0048	0.0052	0.0076	0.0115	0.0010	0.0006	-0.0027	-0.0048	0.0059	-0.0071	-0.0014	-0.0070	-0.0033	0.0021
	**relative bias**	0.0063	0.0775	0.0130	0.0230	-0.0067	0.0532	0.0784	0.2423	0.0064	-0.0123	-0.0764	-0.0119	0.0167	-0.0274	-0.0317	-0.0912	0.2987	0.0722
	**coverage**	96.1	96.3	95.3	95.2	95	95.4	94.8	96.1	94.8	94.8	95.1	96.1	97.5	94.6	95.8	94.3	95.6	95.4

S1: simulation scenario 1, linear proportional effect of age at diagnosis

S2: simulation scenario 2, non-linear non-proportional effect of age, non-proportional effects of categorical stage and deprivation

Annex: Figure1. Simulations

Breast cancer - "true" excess hazard and net survival (black line) and the corresponding simulated effects

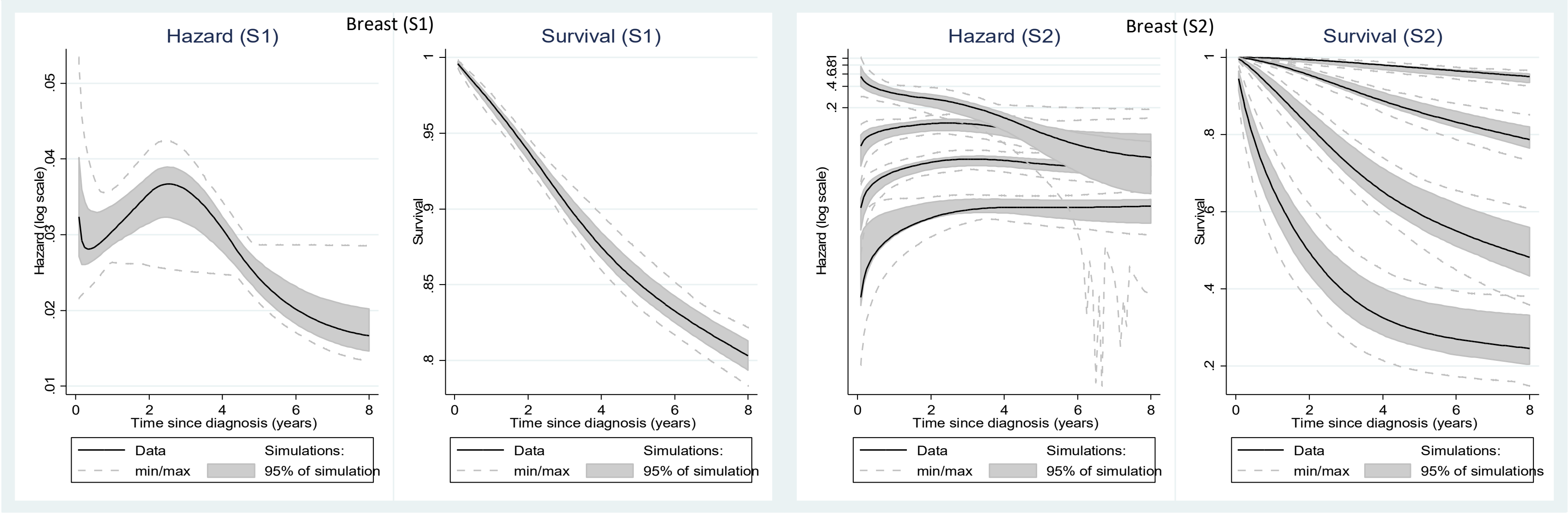

Lung cancer - "true" excess hazard and net survival (black line) and the corresponding simulated effects

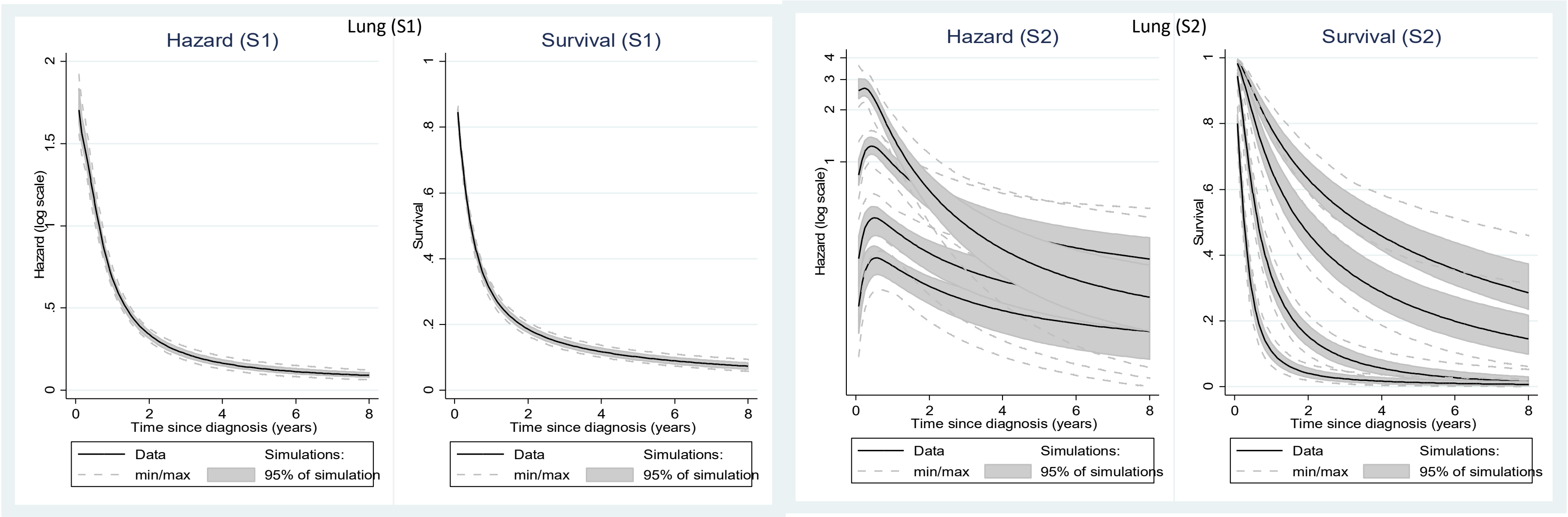

The black curve is the true excess hazard and survival curves

The grey shaded area corresponds to 95% of the simulated hazard and survival curves.

The dashed grey curves correspond to the minimum and maximum values from the 1,000 simulated samples

Annex: Table 2. Estimated average cancer hazard (over 1,000 simulated datasets) at 3 months, 1 year and 5 years after diagnosis from the relative survival and cause-specific approaches

S1: simulation scenario 1, linear proportional effect of age at diagnosis

S2: simulation scenario 2, non-linear non-proportional effect of age, non-proportional effects of categorical stage and deprivation

	3 months				1 year				5 years			
	relative survival approach		cause-specific approach		relative survival approach		cause-specific approach		relative survival approach		cause-specific approach	
	**hazard**	***std dev.***	**hazard**	***std dev.***	**hazard**	***std dev.***	**hazard**	***std dev.***	**hazard**	***std dev.***	**hazard**	***std dev.***
**Breast cancer - S1**												
**All ages**	0.029	*0.005*	0.028	*0.005*	0.030	*0.005*	0.030	*0.004*	0.023	*0.004*	0.023	*0.003*
**15**	0.024	*0.004*	0.024	*0.004*	0.025	*0.003*	0.026	*0.003*	0.020	*0.002*	0.020	*0.002*
**20**	0.025	*0.003*	0.025	*0.004*	0.026	*0.003*	0.026	*0.003*	0.020	*0.002*	0.020	*0.002*
**25**	0.025	*0.003*	0.025	*0.003*	0.027	*0.003*	0.027	*0.003*	0.021	*0.002*	0.021	*0.002*
**30**	0.026	*0.003*	0.026	*0.003*	0.027	*0.003*	0.028	*0.002*	0.021	*0.002*	0.021	*0.002*
**35**	0.027	*0.003*	0.026	*0.003*	0.028	*0.002*	0.028	*0.002*	0.022	*0.002*	0.022	*0.002*
**40**	0.027	*0.003*	0.027	*0.003*	0.028	*0.002*	0.029	*0.002*	0.022	*0.001*	0.022	*0.001*
**45**	0.028	*0.003*	0.027	*0.003*	0.029	*0.002*	0.029	*0.002*	0.023	*0.001*	0.022	*0.001*
**50**	0.028	*0.003*	0.028	*0.003*	0.030	*0.002*	0.030	*0.002*	0.023	*0.001*	0.023	*0.001*
**55**	0.029	*0.003*	0.029	*0.003*	0.030	*0.002*	0.031	*0.002*	0.024	*0.001*	0.023	*0.001*
**60**	0.030	*0.003*	0.029	*0.003*	0.031	*0.002*	0.031	*0.002*	0.024	*0.001*	0.024	*0.001*
**65**	0.031	*0.003*	0.030	*0.003*	0.032	*0.002*	0.032	*0.002*	0.025	*0.001*	0.024	*0.001*
**70**	0.031	*0.003*	0.031	*0.003*	0.033	*0.002*	0.033	*0.002*	0.025	*0.002*	0.025	*0.001*
**75**	0.032	*0.003*	0.031	*0.004*	0.033	*0.002*	0.033	*0.002*	0.026	*0.002*	0.026	*0.002*
**80**	0.033	*0.003*	0.032	*0.004*	0.034	*0.002*	0.034	*0.002*	0.027	*0.002*	0.026	*0.002*
**85**	0.034	*0.004*	0.033	*0.004*	0.035	*0.003*	0.035	*0.003*	0.027	*0.002*	0.027	*0.002*
**90**	0.034	*0.004*	0.033	*0.004*	0.036	*0.003*	0.036	*0.003*	0.028	*0.003*	0.027	*0.002*
**95**	0.035	*0.005*	0.034	*0.005*	0.037	*0.004*	0.036	*0.003*	0.029	*0.003*	0.028	*0.003*
**Lung cancer - S1**												
**All ages**	1.197	*0.559*	1.208	*0.560*	0.545	*0.255*	0.541	*0.251*	0.109	*0.052*	0.110	*0.052*
**15**	0.588	*0.074*	0.596	*0.074*	0.268	*0.033*	0.267	*0.032*	0.053	*0.007*	0.054	*0.007*
**20**	0.640	*0.074*	0.649	*0.073*	0.291	*0.032*	0.291	*0.032*	0.058	*0.007*	0.059	*0.007*
**25**	0.698	*0.073*	0.707	*0.072*	0.317	*0.032*	0.316	*0.031*	0.063	*0.008*	0.064	*0.007*
**30**	0.760	*0.071*	0.769	*0.071*	0.346	*0.031*	0.344	*0.030*	0.069	*0.008*	0.070	*0.007*
**35**	0.828	*0.069*	0.838	*0.069*	0.377	*0.030*	0.375	*0.029*	0.075	*0.008*	0.076	*0.007*
**40**	0.902	*0.066*	0.912	*0.065*	0.411	*0.028*	0.408	*0.028*	0.082	*0.008*	0.083	*0.008*
**45**	0.983	*0.062*	0.994	*0.062*	0.448	*0.026*	0.445	*0.026*	0.089	*0.008*	0.090	*0.008*
**50**	1.072	*0.057*	1.082	*0.057*	0.488	*0.024*	0.485	*0.024*	0.097	*0.008*	0.099	*0.008*
**55**	1.168	*0.053*	1.179	*0.052*	0.532	*0.022*	0.528	*0.022*	0.106	*0.009*	0.107	*0.008*
**60**	1.274	*0.048*	1.285	*0.048*	0.580	*0.020*	0.575	*0.020*	0.116	*0.009*	0.117	*0.009*
**65**	1.389	*0.046*	1.400	*0.045*	0.632	*0.020*	0.627	*0.019*	0.126	*0.010*	0.127	*0.009*
**70**	1.514	*0.047*	1.526	*0.047*	0.689	*0.021*	0.683	*0.021*	0.137	*0.011*	0.139	*0.010*
**75**	1.651	*0.055*	1.663	*0.054*	0.752	*0.026*	0.745	*0.025*	0.150	*0.012*	0.151	*0.011*
**80**	1.801	*0.069*	1.813	*0.069*	0.820	*0.033*	0.812	*0.032*	0.163	*0.014*	0.165	*0.013*
**85**	1.965	*0.090*	1.977	*0.089*	0.895	*0.043*	0.885	*0.042*	0.178	*0.016*	0.180	*0.015*
**90**	2.143	*0.116*	2.155	*0.116*	0.976	*0.056*	0.965	*0.055*	0.195	*0.019*	0.196	*0.018*
**95**	2.339	*0.149*	2.350	*0.148*	1.065	*0.071*	1.053	*0.070*	0.212	*0.022*	0.214	*0.021*

Annex: Table 2. Estimated average cancer hazard (over 1,000 simulated datasets) at 3 months, 1 year and 5 years after diagnosis from the relative survival and cause-specific approaches

S1: simulation scenario 1, linear proportional effect of age at diagnosis

S2: simulation scenario 2, non-linear non-proportional effect of age, non-proportional effects of categorical stage and deprivation

	**3 months**				**1 year**				**5 years**			
	**relative survival approach**		**cause-specific approach**		**relative survival approach**		**cause-specific approach**		**relative survival approach**		**cause-specific approach**	
	**hazard**	***std dev.***	**hazard**	***std dev.***	**hazard**	***std dev.***	**hazard**	***std dev.***	**hazard**	***std dev.***	**hazard**	***std dev.***
**Breast cancer - S2**												
**All ages**	0.209	*0.351*	0.227	*0.373*	0.150	*0.183*	0.144	*0.169*	0.062	*0.053*	0.074	*0.064*
**15**	0.241	*0.375*	0.255	*0.387*	0.183	*0.215*	0.174	*0.199*	0.083	*0.072*	0.098	*0.084*
**20**	0.207	*0.316*	0.220	*0.328*	0.163	*0.191*	0.157	*0.178*	0.077	*0.063*	0.091	*0.075*
**25**	0.177	*0.267*	0.190	*0.278*	0.146	*0.170*	0.141	*0.160*	0.071	*0.055*	0.084	*0.067*
**30**	0.153	*0.227*	0.165	*0.239*	0.131	*0.152*	0.128	*0.144*	0.067	*0.050*	0.079	*0.061*
**35**	0.134	*0.197*	0.147	*0.209*	0.119	*0.138*	0.117	*0.132*	0.063	*0.045*	0.074	*0.057*
**40**	0.121	*0.176*	0.133	*0.188*	0.109	*0.127*	0.108	*0.122*	0.059	*0.042*	0.069	*0.053*
**45**	0.112	*0.163*	0.125	*0.175*	0.103	*0.119*	0.102	*0.115*	0.057	*0.040*	0.066	*0.050*
**50**	0.109	*0.157*	0.121	*0.170*	0.099	*0.115*	0.099	*0.111*	0.054	*0.038*	0.064	*0.048*
**55**	0.110	*0.158*	0.123	*0.172*	0.099	*0.114*	0.099	*0.111*	0.053	*0.037*	0.062	*0.047*
**60**	0.117	*0.168*	0.131	*0.182*	0.102	*0.117*	0.102	*0.114*	0.052	*0.036*	0.061	*0.046*
**65**	0.131	*0.188*	0.147	*0.204*	0.109	*0.125*	0.108	*0.120*	0.051	*0.035*	0.061	*0.046*
**70**	0.154	*0.219*	0.172	*0.237*	0.121	*0.137*	0.119	*0.130*	0.051	*0.035*	0.062	*0.046*
**75**	0.188	*0.265*	0.208	*0.286*	0.137	*0.153*	0.133	*0.144*	0.051	*0.035*	0.063	*0.046*
**80**	0.236	*0.329*	0.260	*0.355*	0.157	*0.173*	0.152	*0.162*	0.050	*0.036*	0.064	*0.046*
**85**	0.301	*0.415*	0.330	*0.446*	0.183	*0.197*	0.173	*0.181*	0.049	*0.037*	0.064	*0.046*
**90**	0.385	*0.524*	0.422	*0.564*	0.212	*0.224*	0.198	*0.202*	0.050	*0.040*	0.063	*0.047*
**95**	0.489	*0.653*	0.536	*0.706*	0.244	*0.252*	0.222	*0.221*	0.052	*0.042*	0.062	*0.049*
**Lung cancer - S2**												
**All ages**	0.988	*1.091*	0.977	*1.060*	0.509	*0.395*	0.482	*0.379*	0.121	*0.083*	0.130	*0.100*
**15**	0.503	*0.468*	0.492	*0.449*	0.245	*0.179*	0.217	*0.162*	0.056	*0.050*	0.052	*0.046*
**20**	0.536	*0.490*	0.527	*0.473*	0.273	*0.191*	0.246	*0.175*	0.064	*0.051*	0.061	*0.048*
**25**	0.570	*0.515*	0.563	*0.499*	0.304	*0.206*	0.278	*0.189*	0.073	*0.052*	0.071	*0.050*
**30**	0.606	*0.541*	0.602	*0.527*	0.339	*0.223*	0.314	*0.206*	0.084	*0.053*	0.084	*0.054*
**35**	0.645	*0.570*	0.643	*0.557*	0.378	*0.243*	0.353	*0.227*	0.098	*0.056*	0.101	*0.058*
**40**	0.688	*0.604*	0.687	*0.592*	0.420	*0.267*	0.397	*0.251*	0.115	*0.059*	0.120	*0.065*
**45**	0.736	*0.644*	0.737	*0.633*	0.465	*0.294*	0.445	*0.279*	0.133	*0.063*	0.142	*0.073*
**50**	0.792	*0.691*	0.794	*0.680*	0.513	*0.324*	0.495	*0.309*	0.151	*0.069*	0.165	*0.084*
**55**	0.859	*0.749*	0.861	*0.737*	0.563	*0.355*	0.546	*0.340*	0.168	*0.076*	0.186	*0.095*
**60**	0.940	*0.818*	0.942	*0.806*	0.613	*0.385*	0.597	*0.372*	0.182	*0.082*	0.204	*0.106*
**65**	1.039	*0.903*	1.041	*0.889*	0.661	*0.414*	0.645	*0.400*	0.189	*0.086*	0.214	*0.113*
**70**	1.163	*1.007*	1.162	*0.989*	0.704	*0.437*	0.687	*0.422*	0.188	*0.086*	0.214	*0.114*
**75**	1.316	*1.133*	1.311	*1.110*	0.739	*0.451*	0.717	*0.435*	0.177	*0.083*	0.201	*0.109*
**80**	1.502	*1.284*	1.490	*1.253*	0.762	*0.457*	0.733	*0.438*	0.156	*0.080*	0.178	*0.100*
**85**	1.722	*1.461*	1.699	*1.418*	0.771	*0.456*	0.733	*0.432*	0.132	*0.076*	0.148	*0.091*
**90**	1.976	*1.663*	1.934	*1.603*	0.763	*0.448*	0.714	*0.419*	0.113	*0.071*	0.123	*0.081*
**95**	2.257	*1.886*	2.186	*1.804*	0.735	*0.441*	0.672	*0.407*	0.095	*0.066*	0.099	*0.072*

Annex: Table 3. Coefficients (average over 1,000 samples) estimated in relative survival and cause-specific models

Effect of age

	**Relative survival approach**		**Cause-specific approach**		**Relative survival approach**		**Cause-specific approach**	
	coeff. linear age	*std dev.*	coeff. linear age	*std dev.*	coeff. non-linear age	*std dev.*	coeff. non-linear age	*std dev.*
**Breast - S1**								
proportional	0.005	*0.003*	0.004	*0.002*				
**Breast - S2**								
proportional	0.252	*0.051*	0.247	*0.048*	-0.185	*0.043*	-0.176	*0.041*
non-proportional	-0.085	*0.025*	-0.084	*0.023*	0.043	*0.021*	0.041	*0.020*
**Lung - S1**								
proportional	0.017	*0.002*	0.017	*0.002*				
**Lung - S2**								
proportional	0.198	*0.023*	0.196	*0.023*	-0.015	*0.023*	-0.013	*0.023*
non-proportional	-0.049	*0.013*	-0.048	*0.012*	0.032	*0.011*	0.035	*0.012*

Effect of stage

	**Relative survival approach**		**Cause-specific approach**		**Relative survival approach**		**Cause-specific approach**		**Relative survival approach**		**Cause-specific approach**	
	coeff. stage 1	*std dev.*	coeff. stage 1	*std dev.*	coeff. stage 2	*std dev.*	coeff. stage 2	*std dev.*	coeff. stage 3	*std dev.*	coeff. stage 3	*std dev.*
**Breast - S1**												
proportional												
**Breast - S2**												
proportional	-4.898	*0.364*	-5.356	*0.333*	-3.304	*0.146*	-3.114	*0.139*	-1.670	*0.143*	-1.558	*0.131*
non-proportional	0.822	*0.173*	0.963	*0.157*	0.748	*0.072*	0.633	*0.070*	0.494	*0.074*	0.430	*0.067*
**Lung - S1**												
proportional												
**Lung - S2**												
proportional	-2.248	*0.115*	-2.204	*0.113*	-1.715	*0.112*	-1.670	*0.109*	-0.709	*0.053*	-0.710	*0.054*
non-proportional	0.399	*0.060*	0.379	*0.068*	0.357	*0.058*	0.333	*0.063*	0.253	*0.033*	0.255	*0.035*

Effect of deprivation

	**Relative survival**		**Cause-specific**		**Relative survival**		**Cause-specific**		**Relative survival**		**Cause-specific**		**Relative survival**		**Cause-specific**	
	coeff. dep 2	*std dev.*	coeff. dep 2	*std dev.*	coeff. dep 3	*std dev.*	coeff. dep 3	*std dev.*	coeff. dep 4	*std dev.*	coeff. dep 4	*std dev.*	coeff. dep 5	*std dev.*	coeff. dep 5	*std dev.*
**Breast - S1**																
proportional																
**Breast - S2**																
proportional	0.263	*0.170*	0.296	*0.156*	0.345	*0.166*	0.326	*0.158*	0.116	*0.186*	0.089	*0.175*	0.378	*0.184*	0.320	*0.170*
non-proportional	-0.109	*0.082*	-0.113	*0.075*	-0.134	*0.081*	-0.102	*0.077*	-0.093	*0.086*	-0.042	*0.081*	-0.092	*0.083*	-0.076	*0.076*
**Lung - S1**																
proportional																
**Lung - S2**																
proportional	0.103	*0.080*	0.099	*0.079*	0.104	*0.081*	0.100	*0.080*	0.059	*0.074*	0.049	*0.072*	0.159	*0.071*	0.160	*0.069*
non-proportional	0.041	*0.043*	0.044	*0.043*	0.070	*0.045*	0.079	*0.045*	-0.014	*0.039*	-0.012	*0.039*	0.031	*0.038*	0.032	*0.039*

S1: simulation scenario 1, linear proportional effect of age at diagnosis
S2: simulation scenario 2, non-linear non-proportional effect of age, non-proportional effects of categorical stage and deprivation
